# Sequence-type diversity of invasive Streptococcus pneumoniae isolates in Iran among children under 15 years of age

**DOI:** 10.3205/dgkh000445

**Published:** 2023-09-07

**Authors:** Sedigheh Rafiei Tabatabaei, Marjan Tariverdi, Abdollah Karimi, Ali Nazari-Alam, Hannan Khodaei, Leila Azimi

**Affiliations:** 1 Pediatric Infections Research Center, Research Institute of Children’s Health, Shahid Beheshti University of Medical Sciences, Tehran, Iran; 2 Department of Pediatric Infectious Disesease, Children’s Clinical Research Development Center, Faculty of Medicine, Hormozgan University of Medical Sciences, Bandar Abbas, Iran; 3 Infectious Diseases Research Center, Kashan University of Medical Sciences, Kashan, Iran

**Keywords:** bacterial meningitis, children, Iran, S. pneumoniae, cerebrospinal fluid, multi-locus sequence typing, sequence types, clonal complexes

## Abstract

**Background:**

Infection with viruses, bacteria, or other pathogens can lead to inflammation of the meninges. Finding the pathogen and identifying the most common type is necessary for each country. Using multi-locus sequence typing (MLST), the aim of this study was to determine the genetic relationship among *S. pneumoniae* isolated from CSF in children with bacterial meningitis.

**Materials and methods:**

: Fourteen isolates of *S. pneumoniae* from CSF in children with bacterial meningitis were included in this study. The seven housekeeping genes, primer, and analysis of the sequencing used in MLST were extracted from PubMLST.

**Results:**

The sequencing analysis showed four MLST types in the studied strains. The most frequent type is ST13649 and the least frequent are ST708 and ST285.

**Conclusion:**

Finding the bacterial sequence types (ST) enables comparing the ST in different, especially neighbouring, countries.

## Zusammenfassung

**Hintergrund:**

Eine Infektion mit Viren, Bakterien oder anderen Erregern kann zur Meningitis führen. Die Suche nach dem Erreger und die Bestimmung des häufigsten Typs ist für jedes Land notwendig. Ziel dieser Studie war es, die genetische Verwandtschaft von aus dem Liquor von Kindern mit bakterieller Meningitis isolierten *S. pneumoniae* durch multi-locus sequence typing (MLST) zu erkennen.

**Materialien und Methoden:**

14 aus Liquor von Kindern mit bakterieller Meningitis isolierte *S. pneumoniae* wurden in die Studie aufgenommen. Die sieben Housekeeping-Gene, Primer und die Analyse der im MLST verwendeten Sequenzierung wurden aus PubMLST extrahiert.

**Ergebnisse:**

Die Sequenzierungsanalyse ergab vier MLST-Typen. Der häufigste Typ war ST13649; ST708 und ST285 traten selten auf.

**Schlussfolgerung:**

Die Ermittlung der bakteriellen ST bietet die Möglichkeit, die bakteriellen Sequenztypen in verschiedenen und insbesondere in angrenzenden Ländern zu vergleichen.

## Introduction

Meningitis is an acute inflammation of the protective membranes covering the brain and spinal cord [1]. The most common symptoms include fever, headache, and neck stiffness; other symptoms have been reported, such as confusion or loss of consciousness, vomiting, and inability to tolerate light or loud noise [2]. Inflammation of the meninges may be due to infection with viruses, bacteria, or other pathogens, but is rarely caused by certain medications [3]. Due to the proximity of inflammation to the brain and spinal cord, meningitis can be dangerous and is therefore classified as a medical emergency [4]. Almost all human pathogenic microorganisms can cause meningitis, but some pathogens, e.g., *H. influenzae*, *S. pneumoniae* and *N. meningitidis*, are the major cause of acute bacterial meningitis reported in infants and children. The reasons for this association are not yet fully understood [5], [6]. Rapid diagnosis and identification of infectious agents in clinical specimens is necessary in order to implement appropriate therapeutic measures. Therefore, an ideal diagnostic test should be sensitive, specific, and rapid in order to maximum the patient's chances of recovery and reduce the incidence of clinical complications [5]. 

Although polymerase chain reaction (PCR) and more recently reverse-transcriptase (RT)-PCR have made an enormous difference in the diagnosis of infectious diseases by reducing the time of diagnosis and increasing diagnostic sensitivity. These methods are not sufficient to help understand the pathogenesis of microorganisms, genomic changes of these organisms or epidemiology of them [7]. In this regard, molecular epidemiological tools such as multi-locus sequence typing (MLST), next generation sequencing (NGS), and pulse-field-gel-electrophoresis (PFGE) can assist the researcher [7]. MLST, a method with high differentiation power, is based on the analysis of nucleotide polymorphisms [8]. MLST analysis of the most common meningitis-causing bacteria from different hosts can increase knowledge of the epidemiology of these infections, and reveal the patterns of transmission and evolution of these microbes [8]. On the other hand, comparing the common types of these bacteria in different countries provides an opportunity for future studies to examine the ways in which these bacteria cross international borders, given the similarities between species in these countries [8]. Implementing MLST, this study aimed to understand the genetic relationship among *S. pneumoniae* strains isolated from cerebrospinal fluid (CSF) in children with bacterial meningitis.

## Materials and methods

Fourteen *S. pneumoniae* isolates from CSF in children under the age of 15 years with bacterial meningitis were included in this study. The bacterial strains were conserved in at –80°C in a freezer at the Pediatric Infections Research Center (PIRC), Shahid Beheshti University of Medical Sciences, Tehran, Iran. The seven housekeeping genes, primer and analysis of the sequencing used in MLST typing were extracted from PubMLST (https://pubmlst.org/organisms/staphylococcus-aureus/primers). These housekeeping genes and primers are shown in Table 1 (Tab. 1).

## Results

A comparison of the present results with the global pneumococcal MLST database by performing eBURST analysis showed that our strains belonged to five different clonal complexes. The sequencing analysis confirmed four MLST types in the studied strains (Table 2 (Tab. 2)). The most frequent type is ST13649; ST708 and ST285 are rare. 

## Discussion

The source of infections can be determined and the follow-up of hospital infection control measures achieved by molecular epidemiology techniques. Furthermore, the spread and frequency of different bacterial clones in different geographical areas can be monitored using molecular epidemiology techniques. In addition, comparisons can be made and the transfer of these clones across countries can also be followed by molecular epidemiology. Some assays, e.g., PFGE, can identify and differentiate between bacterial clones, but the results can only be used locally, because the global DNA sequencing bank for compare the results of studies is not exist for it. While the results of some molecular epidemiology methods like MLST, which was used in this study, can be compared with other results. The ST13649 was the most comment clone of *S. pneumoniae* in our study. In another study in Iran in 2020, ST9533 was the most frequent strain of pneumococcal invasive isolates found in children and adult [9]. Three STs – ST11618, ST14184, ST15253 – were identified in a study on invasive pneumococcal disease (IPD) isolates in Lebanon in 2021 [10]. The different STs detected in the current study and those studies [9], [10] can because of the different age groups in them. On the other hand, in both recent studies [9], [10] the blood and other sterile body fluids samples added to study but we just used *S. pneumoniae* isolated from CSF. 

Thirty-six different STs in IPD strains were identified from 24 centers in 17 provinces in Turkey in 2016. ST242 was found to have the most repetitions [11]. Although Turkey is our neighboring country, we did not have similar STs in our IPD isolates. This might be due to the fact that the two studies were conducted in two different years; perhaps there were different IPD clones existed each year.

In a study in China in 2017, the predominant STs were ST271, ST320, ST876, ST3173, ST236, ST81 and ST342 [12]. ST 156 is cause of IPD in Spain and its spread in south-western Europe [13]. Other *S. pneumoniae* ST types have been reported from different countries, including ST810 and ST13673 and ST3040 in the USA [13], ST6904 in Papua New Guinea and Fiji [1], and ST53 in Denmark [14]. It is thus apparent that different STs of *S. pneumoniae* have been observed globally [12], [15], [13], [14], and the frequency of the different STs of IPD depends on several factors. 

## Conclusions

The determination of the bacterial ST can provide the opportunity to compare the results and common bacterial ST in different countries, especially in bordering countries. This enables researchers to follow the spread of different bacterial clones and identify the source of infection and transmission route. 

## Notes

### Competing interests

The authors declare that they have no competing interests.

### Funding

We are grateful to the entire staff in the Department of Pediatric Infectious Research Center (PIRC), Mofid Children Hospital, Shahid Beheshti University of Medical Sciences, Tehran, Iran (IR.SBMU.RETECH.REC.1396.545).

## Figures and Tables

**Table 1:**
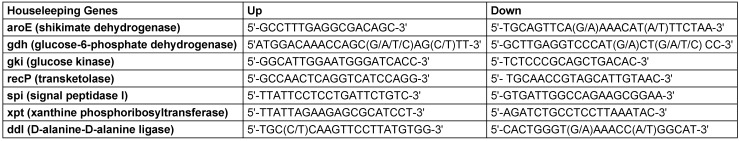
Table1: Housekeeping genes and primers in MLST

**Table 2:**
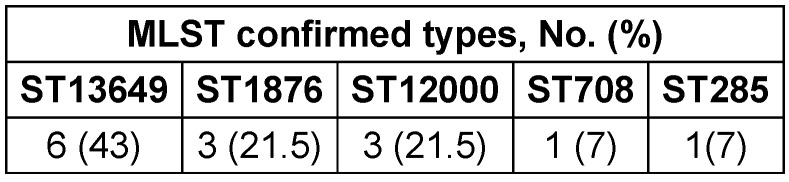
MLST types
